# Crystal structure of zymonic acid and a redetermination of its precursor, pyruvic acid

**DOI:** 10.1107/S2056989019007072

**Published:** 2019-05-24

**Authors:** Dominik Heger, Alexis J. Eugene, Sean R. Parkin, Marcelo I. Guzman

**Affiliations:** a Department of Chemistry, University of Kentucky, Lexington, Kentucky 40506, USA; b Department of Chemistry, Faculty of Science, Masaryk University, Kamenice 5, 625 00 Brno, Czech Republic

**Keywords:** crystal structure, hydrogen bonding, low temperature, zymonic, pyruvic

## Abstract

The structure of zymonic acid (systematic name: 4-hy­droxy-2-methyl-5-oxo-2,5-di­hydro­furan-2-carb­oxy­lic acid), C_6_H_6_O_5_, which had previously eluded crystallographic determination, is presented here for the first time. It forms by intra­molecular condensation of parapyruvic acid, which is the product of aldol condensation of pyruvic acid. A redetermination of the crystal structure of pyruvic acid (systematic name: 2-oxo­propanoic acid), C_3_H_4_O_3_, at low temperature (90 K) and with increased precision, is also presented [for the previous structure, see: Harata *et al.* (1977 ▸). *Acta Cryst.* B**33**, 210–212]. In zymonic acid, the hy­droxy­lactone ring is close to planar (r.m.s. deviation = 0.0108 Å) and the dihedral angle between the ring and the plane formed by the bonds of the methyl and carb­oxy­lic acid carbon atoms to the ring is 88.68 (7)°. The torsion angle of the carb­oxy­lic acid group relative to the ring is 12.04 (16)°. The pyruvic acid mol­ecule is almost planar, having a dihedral angle between the carb­oxy­lic acid and methyl-ketone groups of 3.95 (6)°. Inter­molecular inter­actions in both crystal structures are dominated by hydrogen bonding. The common *R*_2_^2^(8) hydrogen-bonding motif links carb­oxy­lic acid groups on adjacent mol­ecules in both structures. In zymonic acid, this results in dimers about a crystallographic twofold of space group *C*2/*c*, which forces the carb­oxy­lic acid group to be disordered exactly 50:50, which scrambles the carbonyl and hydroxyl groups and gives an apparent equalization of the C—O bond lengths [1.2568 (16) and 1.2602 (16) Å]. The other hydrogen bonds in zymonic acid (O—H⋯O and weak C—H⋯O), link mol­ecules across a 2_1_-screw axis, and generate an *R*_2_^2^(9) motif. These hydrogen-bonding inter­actions propagate to form extended pleated sheets in the *ab* plane. Stacking of these zigzag sheets along *c* involves only van der Waals contacts. In pyruvic acid, inversion-related mol­ecules are linked into *R*_2_^2^(8) dimers, with van der Waals inter­actions between dimers as the only other inter­molecular contacts.

## Abstract 2

Inter­molecular inter­actions in both crystal structures are dominated by hydrogen bonding. The common 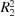
(8) hydrogen-bonding motif links carb­oxy­lic acid groups on adjacent mol­ecules in both structures.

## Chemical context   

The Human Metabolome Database (Wishart *et al.*, 2007 ▸, 2009 ▸, 2013 ▸, 2018 ▸) lists the compound 4-hy­droxy-2-methyl-5-oxo­furan-2-carb­oxy­lic acid (C_6_H_6_O_5_), commonly named zymonic acid, with the metabocard HMDB0031210. Zymonic acid is used as a flavor constituent for confectionery and tobacco products (Yannai, 2004 ▸). The generation of zymonic acid can proceed by condensation of parapyruvic acid, which itself forms by aldol condensation of pyruvic acid (IUPAC name 2-oxo­propanoic acid, C_3_H_4_O_3_; Bloomer *et al.*, 1970 ▸). Therefore, zymonic acid is directly derived from pyruvic acid, and is thus related to the compounds present in the tri­carb­oxy­lic acid (Krebs) cycle (Nelson & Cox, 2004 ▸) and its reductive version (Guzman, 2011 ▸; Guzman & Martin, 2008 ▸; Zhou & Guzman, 2016 ▸). As an inter­mediate in central metabolism, zymonic acid is produced in the cytoplasm at very low concentration, from where it can be excreted to the extracellular region.
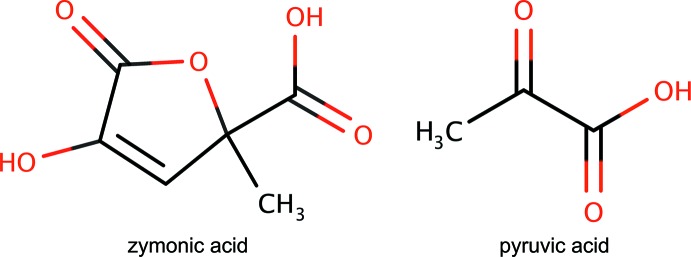


The electron-impact mass spectrum (MS) and electrospray ionization fragmentation of zymonic acid following gas and liquid chromatography, respectively, have been reported (Allen *et al.*, 2015 ▸, 2016 ▸). The use of ^13^C-zymonic acid has enabled mapping of pH changes, independently of concentration, in mammalian organs and tumors *via* hyperpolarized magnetic resonance (Düwel *et al.*, 2017 ▸). Thus, zymonic acid is a non-invasive extracellular imaging sensor to localize and qu­antify pH *in vivo* (Düwel *et al.*, 2017 ▸; Hundshammer *et al.*, 2017 ▸), with many possible applications in medical diagnosis (Schilling *et al.*, 2016 ▸). As part of the process resulting in the aforementioned invention, the detailed ^1^H and ^13^C NMR spectra of pure zymonic acid have been reported (Hundshammer *et al.*, 2017 ▸). Herein, we contribute new information to characterize zymonic acid by reporting for the first time its crystal structure, along with a low-temperature redetermination of pyruvic acid.

## Structural commentary   

Aside from the effects on the geometry of the carb­oxy­lic acid group in zymonic acid that stem from disorder about the twofold axis (see below), there are no unusual bond lengths or angles in either compound.

**Figure 1:**
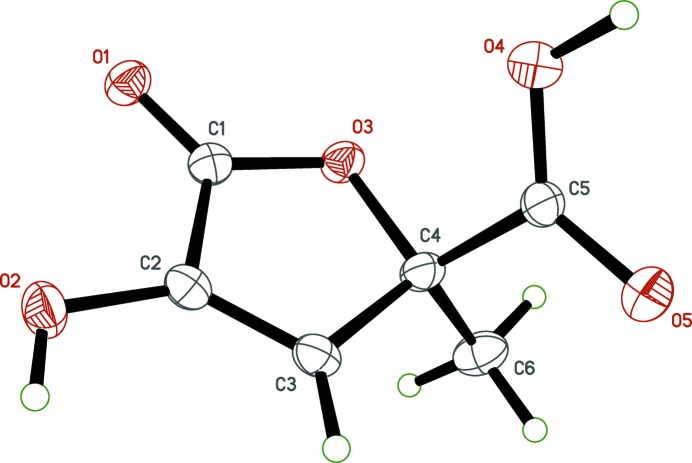
The mol­ecular structure of zymonic acid, with displacement ellipsoids drawn at the 50% probability level.

In zymonic acid (Fig. 1 ▸), the hy­droxy­lactone ring is essentially planar (r.m.s. deviation = 0.0108 Å), with the largest deviation from planarity [0.0171 (8) Å] for the ring oxygen atom, O3. The plane defined by the ring carbon atom C4, the methyl carbon atom C6, and the carb­oxy­lic acid carbon atom C5, is almost perpendicular to the mean plane of the ring atoms [dihedral angle = 88.68 (7)°]. Lastly, the orientation of the carb­oxy­lic acid group relative to the ring, as defined by the torsion angle O4—C5—C4—O3, is 12.04 (16)°. For the carb­oxy­lic acid group, disorder about the crystallographic twofold axis effectively averages the C=O double and C—O single bonds, rendering them equivalent [the C5—O4 and C5—O5 distances are 1.2568 (16) and 1.2602 (16) Å, respectively], and requires modeling of half-occupancy hydrogens (H4*O* and H5*O*) on each.

**Figure 2:**
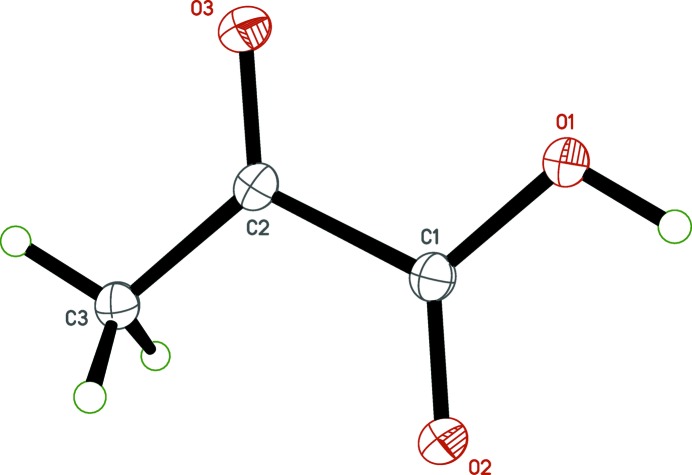
The mol­ecular structure of pyruvic acid, with displacement ellipsoids drawn at the 50% probability level.

In spite of increased precision resulting from much lower temperature (90 K *versus* 266 K) and data collection on modern equipment, the redetermined structure of pyruvic acid (Fig. 2 ▸) is largely unchanged from that reported by Harata *et al.* (1977 ▸). For example, the dihedral angle between the planes defined by atoms C1/C2/C3/O3 and C1/C2/O1/O2 is 3.95 (6)° at 90.00 (2) K *versus* 3.5° at 266 (1) K.

## Supra­molecular features   

**Figure 3:**
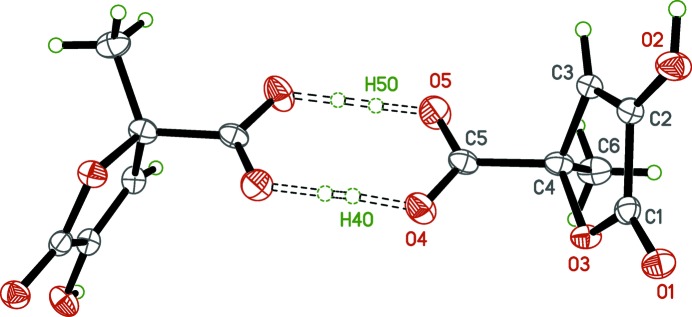
The 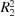
(8) dimer of zymonic acid. Unlabeled atoms are related to their labeled counterparts by a crystallographic twofold axis (1 − *x*, *y*, 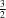 − *z*). This uncommon symmetry [for an 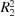
(8) dimer] forces the O—H⋯O hydrogen bonds involved to be 50:50 disordered about the twofold axis.

**Figure 4:**
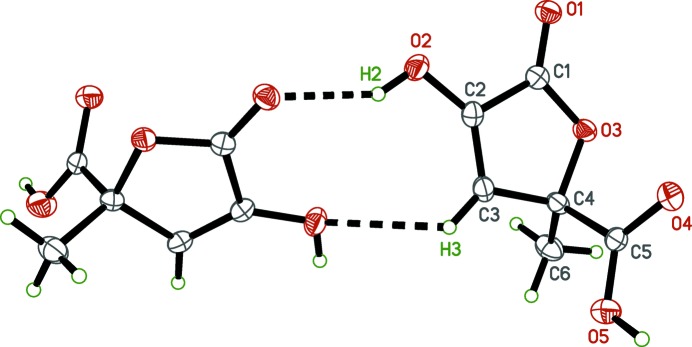
The 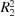
(9) dimer of zymonic acid. Unlabeled atoms are related to their labeled counterparts by a crystallographic 2_1_-screw axis (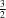 − *x*, 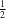 + *y*, 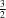 − *z*). Disorder of the carb­oxy­lic acid H atoms is omitted to enhance clarity.

**Figure 5:**
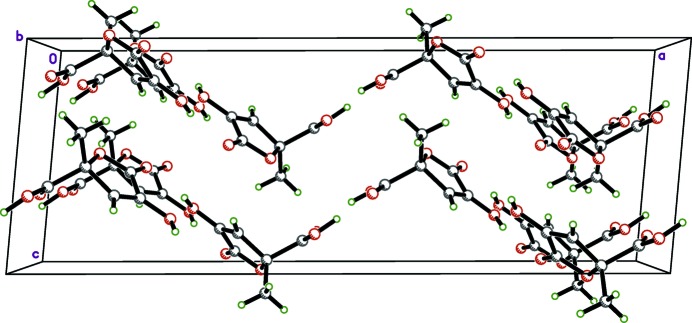
A packing plot of zymonic acid viewed down the *b* axis, showing the stacking along *c* of zigzag pleated assemblies of mol­ecules. Disorder of the carb­oxy­lic acid hydrogen atoms is omitted to enhance clarity.

**Figure 6:**
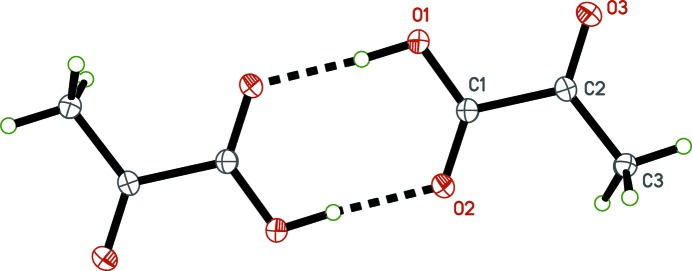
The 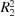
(8) dimer of pyruvic acid. Unlabeled atoms are related to their labeled counterparts by crystallographic inversion symmetry (1 − *x*, 1 − *y*, 1 − *z*).

The main inter­molecular inter­actions in the crystals of both zymonic and pyruvic acids are hydrogen bonds. In zymonic acid, the carb­oxy­lic acid groups of adjacent mol­ecules are related by a crystallographic twofold axis to form hydrogen bonds [O4—H4*O*⋯O4^ii^ and O5—H5*O*⋯O5^ii^; symmetry code: (ii) 1 − *x*, *y*, 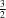 − *z*] giving 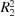
(8) dimer motifs (Table 1 ▸). This common supra­molecular construct in carb­oxy­lic acids usually occurs between inversion-related or symmetry-independent mol­ecules. Here, the orientation of the dimer relative to the crystallographic twofold axis forces the average structure to be statistically disordered (Fig. 3 ▸). Another pair of hydrogen bonds [O2—H2⋯O1^i^ and C3—H3⋯O2^i^; symmetry code: (i) 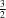 − *x*, 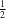 + *y*, 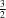 − *z*], link mol­ecules related by a 2_1_-screw axis, into 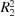
(9) motifs (Fig. 4 ▸). These hydrogen-bonding inter­actions combine to form extended pleated sheets that propagate in the *ab* plane (Fig. 5 ▸), which in turn, stack along the *c*-axis direction. In pyruvic acid, inversion-related mol­ecules form the common 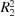
(8) dimer motif (Fig. 6 ▸, Table 2 ▸). In accordance with the work of Harata *et al.* (1977 ▸), there are no other noteworthy inter­molecular inter­actions.

## Database survey   

A search of the Cambridge Crystal Structure Database (Version 5.40, Nov. 2018; Groom *et al.*, 2016 ▸) for zymonic acid gave no hits for searches on either ‘zymonic’ or on the structural formula. A search on the structural formula of pyruvic acid gave two hits. CSD entry PRUVAC (Harata *et al.*, 1977 ▸) describes the pure compound at 266 K, and is similar to the present pyruvic acid structure (after transformation to a common cell setting). CSD entry FAFGUR (Prohens *et al.*, 2016 ▸) describes a co-crystal of pyruvic acid with the drug agomelatine. The CSD does contain structures for derivatives of both zymonic and pyruvic acids, but none of these have features that are especially relevant to the current work.

## Synthesis and crystallization   

Vacuum distillation of pyruvic acid (Sigma–Aldrich, 98.5%) was used for purification (Eugene & Guzman, 2017*a* ▸,*b* ▸). Freshly distilled pyruvic acid was crystallized in a closed vial in a freezer at 253 K. The tail of this distillation, a viscous yellowish residue enriched in parapyruvic and zymonic acids, was isolated in a vial, and the headspace filled with N_2_(g) before sealing it with a cap. Crystals of zymonic acid were produced slowly from this isolated residue kept at 275 K inside a refrigerator. The easily identifiable transparent crystals of zymonic acid appear above the level of the viscous solution within two weeks. Pyruvic acid crystals are deliquescent in air, even at 263 K (Harata *et al.*, 1977 ▸), so they had to be kept cold, with minimal exposure to ambient air. Thus, throughout all experimental stages from initial inspection through data collection, special techniques for crystal handling at low temperature (Parkin & Hope, 1998 ▸) were employed.

## Refinement   

Crystal data, data collection, and structure refinement details are summarized in Table 3 ▸. Non-disordered hydrogen atoms were found in difference Fourier maps. For pyruvic acid, the hydroxyl hydrogen-atom coordinates were refined freely, while methyl hydrogen C—H distances used a riding model that allowed the C—H distance to refine. For zymonic acid, riding models were used for all hydrogen atoms apart from those disordered about the twofold axis, which were modeled in accordance with the recommendations of Fábry (2018 ▸). *U*_iso_(H) parameters of non-disordered hydrogens were set to either 1.2*U*_eq_ or 1.5*U*_eq_ (for the methyl and hydrox­yl groups, respectively) of the attached atom. To ensure stable refinement of disordered groups in the zymonic acid structure, constraints (*SHELXL* command EADP) were used to equalize displacement parameters of superimposed atoms.

## Supplementary Material

Crystal structure: contains datablock(s) global, pyruvic, zymonic. DOI: 10.1107/S2056989019007072/hb7818sup1.cif

Structure factors: contains datablock(s) zymonic. DOI: 10.1107/S2056989019007072/hb7818zymonicsup2.hkl

Structure factors: contains datablock(s) pyruvic. DOI: 10.1107/S2056989019007072/hb7818pyruvicsup3.hkl

Supporting information file. DOI: 10.1107/S2056989019007072/hb7818zymonicsup4.cml

Supporting information file. DOI: 10.1107/S2056989019007072/hb7818pyruvicsup5.cml

CCDC references: 1916323, 1916322

Additional supporting information:  crystallographic information; 3D view; checkCIF report

## Figures and Tables

**Table 1:** Hydrogen-bond geometry (Å, °) for zymonic acid[Chem scheme1]

*D*—H⋯*A*	*D*—H	H⋯*A*	*D*⋯*A*	*D*—H⋯*A*
O2—H2⋯O1^i^	0.84	1.96	2.7103 (14)	148
C3—H3⋯O2^i^	0.95	2.48	3.0720 (16)	120
O4—H4*O*⋯O4^ii^	1.09	1.52	2.607 (2)	176
O5—H5*O*⋯O5^ii^	0.99	1.63	2.624 (2)	179

Symmetry codes: (i) 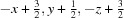; (ii) 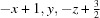.

**Table 2:** Hydrogen-bond geometry (Å, °) for pyruvic acid[Chem scheme1]

*D*—H⋯*A*	*D*—H	H⋯*A*	*D*⋯*A*	*D*—H⋯*A*
O1—H1⋯O2^i^	0.913 (14)	1.742 (14)	2.6536 (8)	175.5 (12)

Symmetry code: (i) 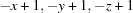.

**Table 3:** Experimental details

	zymonic acid	pyruvic acid
Crystal data		
Chemical formula	C_6_H_6_O_5_	C_3_H_4_O_3_
*M*_r_	158.11	88.06
Crystal system, space group	Monoclinic, *C*2/*c*	Monoclinic, *P*2_1_/*c*
Temperature (K)	90	90
*a*, *b*, *c* (Å)	24.145 (3), 6.6523 (7), 8.6201 (7)	10.7486 (3), 5.1925 (2), 6.8302 (2)
β (°)	95.169 (4)	99.063 (1)
*V* (Å^3^)	1378.9 (3)	376.45 (2)
*Z*	8	4
Radiation type	Mo *K*α	Mo *K*α
μ (mm^−1^)	0.14	0.14
Crystal size (mm)	0.30 × 0.25 × 0.02	0.26 × 0.22 × 0.18

Data collection		
Diffractometer	Bruker D8 Venture dual source	Bruker D8 Venture dual source
Absorption correction	Multi-scan (*SADABS*; Krause *et al.*, 2015 ▸)	Multi-scan (*SADABS*; Krause *et al.*, 2015 ▸)
*T*_min_, *T*_max_	0.721, 0.959	0.890, 0.971
No. of measured, independent and observed [*I* > 2σ(*I*)] reflections	18595, 1586, 1392	10479, 1425, 1242
*R*_int_	0.062	0.025
(sin θ/λ)_max_ (Å^−1^)	0.650	0.796

Refinement		
*R*[*F* ^2^ > 2σ(*F* ^2^)], *wR*(*F* ^2^), *S*	0.037, 0.100, 1.09	0.031, 0.082, 1.08
No. of reflections	1586	1425
No. of parameters	104	60
H-atom treatment	H atoms treated by a mixture of independent and constrained refinement	H atoms treated by a mixture of independent and constrained refinement
Δρ_max_, Δρ_min_ (e Å^−3^)	0.36, −0.24	0.40, −0.21

Computer programs: *APEX3* (Bruker, 2016 ▸), *SHELXT* (Sheldrick, 2015*a* ▸), *SHELXL2018/3* (Sheldrick, 2015*b* ▸), *XP in *SHELXTL** (Sheldrick, 2008 ▸), and *CIFFIX* (Parkin, 2013 ▸).

## supplementary crystallographic information

### 4-Hydroxy-2-methyl-5-oxo-2,5-dihydrofuran-2-carboxylic acid (zymonic) Crystal data

**Table:**

C_6_H_6_O_5_	*F*(000) = 656
*M**_r_* = 158.11	*D*_x_ = 1.523 Mg m^−^^3^
Monoclinic, *C*2/*c*	Mo *K*α radiation, λ = 0.71073 Å
*a* = 24.145 (3) Å	Cell parameters from 9925 reflections
*b* = 6.6523 (7) Å	θ = 3.2–27.5°
*c* = 8.6201 (7) Å	µ = 0.14 mm^−^^1^
β = 95.169 (4)°	*T* = 90 K
*V* = 1378.9 (3) Å^3^	Thin plate, colourless
*Z* = 8	0.30 × 0.25 × 0.02 mm

### 4-Hydroxy-2-methyl-5-oxo-2,5-dihydrofuran-2-carboxylic acid (zymonic) Data collection

**Table:**

Bruker D8 Venture dual source diffractometer	1586 independent reflections
Radiation source: microsource	1392 reflections with *I* > 2σ(*I*)
Detector resolution: 5.6 pixels mm^-1^	*R*_int_ = 0.062
φ and ω scans	θ_max_ = 27.5°, θ_min_ = 3.2°
Absorption correction: multi-scan (*SADABS*; Krause *et al.*, 2015)	*h* = −31→31
*T*_min_ = 0.721, *T*_max_ = 0.959	*k* = −8→8
18595 measured reflections	*l* = −11→10

### 4-Hydroxy-2-methyl-5-oxo-2,5-dihydrofuran-2-carboxylic acid (zymonic) Refinement

**Table:**

Refinement on *F*^2^	Secondary atom site location: difference Fourier map
Least-squares matrix: full	Hydrogen site location: mixed
*R*[*F*^2^ > 2σ(*F*^2^)] = 0.037	H atoms treated by a mixture of independent and constrained refinement
*wR*(*F*^2^) = 0.100	*w* = 1/[σ^2^(*F*_o_^2^) + (0.0428*P*)^2^ + 1.4377*P*] where *P* = (*F*_o_^2^ + 2*F*_c_^2^)/3
*S* = 1.09	(Δ/σ)_max_ = 0.001
1586 reflections	Δρ_max_ = 0.36 e Å^−^^3^
104 parameters	Δρ_min_ = −0.24 e Å^−^^3^
0 restraints	Extinction correction: SHELXL2018 (Sheldrick, 2015a), Fc^*^=kFc[1+0.001xFc^2^λ^3^/sin(2θ)]^-1/4^
Primary atom site location: structure-invariant direct methods	Extinction coefficient: 0.0057 (13)

### 4-Hydroxy-2-methyl-5-oxo-2,5-dihydrofuran-2-carboxylic acid (zymonic) Special details

**Table:**

Experimental. The crystal was mounted using polyisobutene oil on the tip of a fine glass fibre, which was fastened in a copper mounting pin with electrical solder. It was placed directly into the cold gas stream of a liquid-nitrogen based cryostat (Hope, 1994; Parkin & Hope, 1998). Diffraction data were collected with the crystal at 90K, which is standard practice in this laboratory for the majority of flash-cooled crystals.
Geometry. All esds (except the esd in the dihedral angle between two l.s. planes) are estimated using the full covariance matrix. The cell esds are taken into account individually in the estimation of esds in distances, angles and torsion angles; correlations between esds in cell parameters are only used when they are defined by crystal symmetry. An approximate (isotropic) treatment of cell esds is used for estimating esds involving l.s. planes.
Refinement. Refinement progress was checked using *Platon* (Spek, 2009) and by an *R*-tensor (Parkin, 2000). The final model was further checked with the IUCr utility *checkCIF*.

### 4-Hydroxy-2-methyl-5-oxo-2,5-dihydrofuran-2-carboxylic acid (zymonic) Fractional atomic coordinates and isotropic or equivalent isotropic displacement parameters (Å^2^)

**Table:**

	*x*	*y*	*z*	*U*_iso_*/*U*_eq_	Occ. (<1)
C1	0.67036 (5)	0.40914 (19)	0.56905 (14)	0.0170 (3)	
O1	0.68738 (4)	0.24154 (14)	0.54864 (11)	0.0209 (2)	
C2	0.69378 (5)	0.5659 (2)	0.67706 (14)	0.0178 (3)	
O2	0.74026 (4)	0.51656 (15)	0.76588 (12)	0.0240 (3)	
H2	0.752557	0.618162	0.815574	0.036*	
C3	0.66149 (5)	0.7275 (2)	0.66335 (15)	0.0182 (3)	
H3	0.667502	0.849554	0.719574	0.022*	
O3	0.62412 (4)	0.48283 (13)	0.48901 (11)	0.0188 (2)	
C4	0.61371 (5)	0.68442 (19)	0.54353 (15)	0.0186 (3)	
O4	0.54061 (4)	0.51162 (15)	0.66013 (12)	0.0249 (3)	
H4O	0.507244	0.517998	0.736346	0.109 (14)*	0.5
O5	0.54177 (4)	0.84824 (16)	0.66262 (14)	0.0303 (3)	
H5O	0.509866	0.849228	0.727610	0.109 (14)*	0.5
C5	0.56056 (5)	0.67919 (19)	0.62725 (15)	0.0188 (3)	
C6	0.60945 (7)	0.8285 (2)	0.40645 (17)	0.0257 (3)	
H6A	0.644218	0.825746	0.355945	0.039*	
H6B	0.602833	0.964985	0.443442	0.039*	
H6C	0.578557	0.787894	0.331471	0.039*	

### 4-Hydroxy-2-methyl-5-oxo-2,5-dihydrofuran-2-carboxylic acid (zymonic) Atomic displacement parameters (Å^2^)

**Table:**

	*U*^11^	*U*^22^	*U*^33^	*U*^12^	*U*^13^	*U*^23^
C1	0.0184 (6)	0.0180 (6)	0.0151 (6)	−0.0007 (5)	0.0038 (5)	0.0010 (5)
O1	0.0238 (5)	0.0173 (5)	0.0213 (5)	0.0015 (4)	0.0010 (4)	−0.0013 (4)
C2	0.0165 (6)	0.0204 (6)	0.0165 (6)	−0.0023 (5)	0.0016 (5)	−0.0020 (5)
O2	0.0186 (5)	0.0249 (5)	0.0274 (5)	0.0035 (4)	−0.0050 (4)	−0.0073 (4)
C3	0.0168 (6)	0.0190 (6)	0.0190 (6)	−0.0032 (5)	0.0020 (5)	−0.0036 (5)
O3	0.0213 (5)	0.0155 (5)	0.0192 (5)	0.0003 (3)	−0.0013 (4)	−0.0027 (3)
C4	0.0205 (6)	0.0135 (6)	0.0212 (6)	−0.0007 (5)	−0.0012 (5)	−0.0018 (5)
O4	0.0225 (5)	0.0207 (5)	0.0314 (6)	−0.0031 (4)	0.0027 (4)	0.0013 (4)
O5	0.0252 (5)	0.0209 (5)	0.0457 (7)	0.0027 (4)	0.0074 (5)	−0.0040 (4)
C5	0.0165 (6)	0.0171 (6)	0.0217 (6)	0.0004 (5)	−0.0042 (5)	−0.0001 (5)
C6	0.0351 (8)	0.0192 (7)	0.0223 (7)	−0.0013 (6)	−0.0008 (6)	0.0026 (5)

### 4-Hydroxy-2-methyl-5-oxo-2,5-dihydrofuran-2-carboxylic acid (zymonic) Geometric parameters (Å, º)

**Table:**

C1—O1	1.2067 (16)	C4—C6	1.5179 (19)
C1—O3	1.3505 (15)	C4—C5	1.5282 (19)
C1—C2	1.4763 (18)	O4—C5	1.2568 (16)
C2—C3	1.3268 (18)	O4—H4O	1.0854
C2—O2	1.3411 (16)	O5—C5	1.2602 (16)
O2—H2	0.8400	O5—H5O	0.9926
C3—C4	1.5051 (17)	C6—H6A	0.9800
C3—H3	0.9500	C6—H6B	0.9800
O3—C4	1.4505 (15)	C6—H6C	0.9800
			
O1—C1—O3	122.52 (12)	O3—C4—C5	108.03 (10)
O1—C1—C2	128.95 (12)	C3—C4—C5	107.73 (11)
O3—C1—C2	108.53 (11)	C6—C4—C5	112.41 (11)
C3—C2—O2	134.75 (12)	C5—O4—H4O	115.0
C3—C2—C1	109.12 (11)	C5—O5—H5O	117.2
O2—C2—C1	116.12 (11)	O4—C5—O5	125.69 (13)
C2—O2—H2	109.5	O4—C5—C4	118.80 (11)
C2—C3—C4	108.35 (11)	O5—C5—C4	115.45 (11)
C2—C3—H3	125.8	C4—C6—H6A	109.5
C4—C3—H3	125.8	C4—C6—H6B	109.5
C1—O3—C4	109.24 (10)	H6A—C6—H6B	109.5
O3—C4—C3	104.7 (1)	C4—C6—H6C	109.5
O3—C4—C6	109.47 (11)	H6A—C6—H6C	109.5
C3—C4—C6	114.07 (11)	H6B—C6—H6C	109.5
			
O1—C1—C2—C3	−179.25 (13)	C1—O3—C4—C5	−112.19 (11)
O3—C1—C2—C3	1.17 (15)	C2—C3—C4—O3	−1.69 (14)
O1—C1—C2—O2	−0.3 (2)	C2—C3—C4—C6	−121.33 (13)
O3—C1—C2—O2	−179.92 (10)	C2—C3—C4—C5	113.14 (12)
O2—C2—C3—C4	−178.23 (14)	O3—C4—C5—O4	12.04 (16)
C1—C2—C3—C4	0.39 (15)	C3—C4—C5—O4	−100.56 (13)
O1—C1—O3—C4	178.13 (12)	C6—C4—C5—O4	132.92 (13)
C2—C1—O3—C4	−2.26 (13)	O3—C4—C5—O5	−170.61 (11)
C1—O3—C4—C3	2.43 (13)	C3—C4—C5—O5	76.79 (14)
C1—O3—C4—C6	125.11 (12)	C6—C4—C5—O5	−49.72 (16)

### 4-Hydroxy-2-methyl-5-oxo-2,5-dihydrofuran-2-carboxylic acid (zymonic) Hydrogen-bond geometry (Å, º)

**Table:**

*D*—H···*A*	*D*—H	H···*A*	*D*···*A*	*D*—H···*A*
O2—H2···O1^i^	0.84	1.96	2.7103 (14)	148
C3—H3···O2^i^	0.95	2.48	3.0720 (16)	120
O4—H4*O*···O4^ii^	1.09	1.52	2.607 (2)	176
O5—H5*O*···O5^ii^	0.99	1.63	2.624 (2)	179

Symmetry codes: (i) −*x*+3/2, *y*+1/2, −*z*+3/2; (ii) −*x*+1, *y*, −*z*+3/2.

### 2-Oxopropanoic acid (pyruvic) Crystal data

**Table:**

C_3_H_4_O_3_	*F*(000) = 184
*M**_r_* = 88.06	*D*_x_ = 1.554 Mg m^−^^3^
Monoclinic, *P*2_1_/*c*	Mo *K*α radiation, λ = 0.71073 Å
*a* = 10.7486 (3) Å	Cell parameters from 6955 reflections
*b* = 5.1925 (2) Å	θ = 3.8–34.3°
*c* = 6.8302 (2) Å	µ = 0.14 mm^−^^1^
β = 99.063 (1)°	*T* = 90 K
*V* = 376.45 (2) Å^3^	Well-facetted block, colourless
*Z* = 4	0.26 × 0.22 × 0.18 mm

### 2-Oxopropanoic acid (pyruvic) Data collection

**Table:**

Bruker D8 Venture dual source diffractometer	1425 independent reflections
Radiation source: microsource	1242 reflections with *I* > 2σ(*I*)
Detector resolution: 5.6 pixels mm^-1^	*R*_int_ = 0.025
φ and ω scans	θ_max_ = 34.5°, θ_min_ = 3.8°
Absorption correction: multi-scan (*SADABS*; Krause *et al.*, 2015)	*h* = −16→16
*T*_min_ = 0.890, *T*_max_ = 0.971	*k* = −7→8
10479 measured reflections	*l* = −10→10

### 2-Oxopropanoic acid (pyruvic) Refinement

**Table:**

Refinement on *F*^2^	Primary atom site location: structure-invariant direct methods
Least-squares matrix: full	Secondary atom site location: difference Fourier map
*R*[*F*^2^ > 2σ(*F*^2^)] = 0.031	Hydrogen site location: mixed
*wR*(*F*^2^) = 0.082	H atoms treated by a mixture of independent and constrained refinement
*S* = 1.08	*w* = 1/[σ^2^(*F*_o_^2^) + (0.0299*P*)^2^ + 0.1264*P*] where *P* = (*F*_o_^2^ + 2*F*_c_^2^)/3
1425 reflections	(Δ/σ)_max_ < 0.001
60 parameters	Δρ_max_ = 0.40 e Å^−^^3^
0 restraints	Δρ_min_ = −0.21 e Å^−^^3^

### 2-Oxopropanoic acid (pyruvic) Special details

**Table:**

Experimental. The crystal was mounted using polyisobutene oil on the tip of a fine glass fibre, which was fastened in a copper mounting pin with electrical solder. It was placed directly into the cold gas stream of a liquid-nitrogen based cryostat (Parkin & Hope, 1998). Diffraction data were collected with the crystal at 90K, which is standard practice in this laboratory for the majority of flash-cooled crystals.
Geometry. All esds (except the esd in the dihedral angle between two l.s. planes) are estimated using the full covariance matrix. The cell esds are taken into account individually in the estimation of esds in distances, angles and torsion angles; correlations between esds in cell parameters are only used when they are defined by crystal symmetry. An approximate (isotropic) treatment of cell esds is used for estimating esds involving l.s. planes.
Refinement. Refinement progress was checked using *Platon* (Spek, 2009) and by an *R*-tensor (Parkin, 2000). The final model was further checked with the IUCr utility *checkCIF*.

### 2-Oxopropanoic acid (pyruvic) Fractional atomic coordinates and isotropic or equivalent isotropic displacement parameters (Å^2^)

**Table:**

	*x*	*y*	*z*	*U*_iso_*/*U*_eq_	
O1	0.41673 (5)	0.30370 (12)	0.30191 (9)	0.01968 (14)	
H1	0.4903 (13)	0.320 (3)	0.3883 (19)	0.030*	
C1	0.33963 (6)	0.48448 (14)	0.34005 (11)	0.01316 (14)	
O2	0.36317 (5)	0.65673 (11)	0.46175 (8)	0.01589 (13)	
C2	0.20853 (6)	0.46732 (14)	0.21028 (10)	0.01271 (14)	
C3	0.11464 (7)	0.65949 (15)	0.25725 (11)	0.01520 (15)	
H3A	0.1499 (3)	0.8314 (12)	0.255 (1)	0.023*	
H3B	0.0940 (5)	0.6245 (9)	0.388 (1)	0.023*	
H3C	0.0389 (6)	0.6478 (9)	0.1594 (9)	0.023*	
O3	0.18824 (5)	0.30508 (11)	0.08250 (9)	0.01774 (14)	

### 2-Oxopropanoic acid (pyruvic) Atomic displacement parameters (Å^2^)

**Table:**

	*U*^11^	*U*^22^	*U*^33^	*U*^12^	*U*^13^	*U*^23^
O1	0.0132 (2)	0.0218 (3)	0.0222 (3)	0.0041 (2)	−0.0027 (2)	−0.0084 (2)
C1	0.0121 (3)	0.0138 (3)	0.0136 (3)	−0.0002 (2)	0.0018 (2)	0.0003 (2)
O2	0.0135 (2)	0.0155 (3)	0.0176 (3)	−0.00038 (19)	−0.00071 (19)	−0.0034 (2)
C2	0.0119 (3)	0.0139 (3)	0.0121 (3)	−0.0011 (2)	0.0010 (2)	0.0009 (2)
C3	0.0140 (3)	0.0163 (3)	0.0148 (3)	0.0023 (2)	0.0008 (2)	−0.0013 (3)
O3	0.0167 (3)	0.0181 (3)	0.0173 (3)	−0.0002 (2)	−0.0008 (2)	−0.0049 (2)

### 2-Oxopropanoic acid (pyruvic) Geometric parameters (Å, º)

**Table:**

O1—C1	1.3053 (9)	C2—C3	1.4896 (10)
O1—H1	0.913 (14)	C3—H3A	0.971 (6)
C1—O2	1.2201 (9)	C3—H3B	0.971 (6)
C1—C2	1.5446 (10)	C3—H3C	0.971 (6)
C2—O3	1.2079 (9)		
			
C1—O1—H1	108.4 (8)	C2—C3—H3A	109.5
O2—C1—O1	126.37 (7)	C2—C3—H3B	109.5
O2—C1—C2	120.38 (6)	H3A—C3—H3B	109.5
O1—C1—C2	113.24 (6)	C2—C3—H3C	109.5
O3—C2—C3	124.85 (7)	H3A—C3—H3C	109.5
O3—C2—C1	119.96 (7)	H3B—C3—H3C	109.5
C3—C2—C1	115.19 (6)		
			
O2—C1—C2—O3	175.81 (7)	O2—C1—C2—C3	−4.59 (10)
O1—C1—C2—O3	−3.34 (10)	O1—C1—C2—C3	176.26 (6)

### 2-Oxopropanoic acid (pyruvic) Hydrogen-bond geometry (Å, º)

**Table:**

*D*—H···*A*	*D*—H	H···*A*	*D*···*A*	*D*—H···*A*
O1—H1···O2^i^	0.913 (14)	1.742 (14)	2.6536 (8)	175.5 (12)

Symmetry code: (i) −*x*+1, −*y*+1, −*z*+1.
